# Promoting anti-corruption, transparency and accountability to achieve universal health coverage

**DOI:** 10.1080/16549716.2019.1700660

**Published:** 2020-03-20

**Authors:** Theadora Koller, David Clarke, Taryn Vian

**Affiliations:** Gender, Equity, Human Rights, World Health Organization, Geneva, Switzerland; Department of Health Systems Governance and Financing, World Health Organization, Geneva, Switzerland; Special Issue Guest Editor, Department of Nursing and Health Professions, University of San Francisco, San Francisco, CA, USA

**Keywords:** Taryn Vian, University of San Francisco, California USA, Anti-Corruption, Transparency and Accountability, Anti-corruption, transparency, accountability, health systems strengthening, SDGs, fraud risk assessment, pharmaceuticals, human resources

Anti-corruption, transparency and accountability measures are often missing from efforts to promote universal health coverage. Yet, if unchecked, corruption represents a significant drain on domestic health resources and a major barrier to achieving universal health coverage and the sustainable development goals. The World Health Organization (WHO) is promoting a coordinated public health approach to anti-corruption, transparency and accountability, working with global partners to create new internal control and assurance models; increase monitoring and evaluation; develop capacity for multiple stakeholders to address corruption; and strengthen normative guidance to integrate anti-corruption, transparency and accountability into WHO’s work on health systems strengthening. The articles in this special issue explore evidence on the impact of corruption on health, human rights approaches to control corruption, corruption in human resources, tools for addressing corruption in the pharmaceutical sector, and solutions to improve transparency and accountability. New approaches to risk assessment are also proposed. Moving forward, this issue represents a call for action to combat health system corruption through targeted research, informed strategies, and effective cross-sectoral interventions. Taking steps now will allow all countries to seize the pledge of leaving no one behind in addressing inequalities and achieving health for all.

Universal health coverage (UHC) is an objective towards which all countries strive. UHC means that all individuals and communities receive the health services they need without suffering financial hardship. Work toward UHC focuses on health system strengthening efforts, policies, and programs that aim to address gaps in financing, service delivery and improve access to health workers, medicines and vaccines. Anti-corruption, transparency and accountability (ACTA) measures are often neglected in these efforts, a critical omission because if unchecked, corruption in health systems represents a significant drain on domestic health resources and poses a major barrier to efforts to the attainment of UHC and other health-related Sustainable Development Goal (SDG) targets. WHO therefore regards ACTA measures as central components of health systems strengthening for UHC and actively supports member states’ ACTA efforts as part of its general program of work.

WHO prioritizes work on ACTA because ACTA measures are essential for upholding the right to health and other indivisible rights which are at the heart of WHO’s mandate. Without ACTA measures, resources meant to deliver on health goals may be wasted, trust in the health system is weakened, and, most importantly, human lives can be lost. Researchers estimate that 1.6% of the world deaths in children, or 140,000 child deaths per year, could be indirectly attributed to corruption [1]. Because corruption in the health sector has a disproportionate effect on disadvantaged populations, and is a major driver of health inequities, WHO considers that ACTA measures are essential for ensuring that ‘no one is to be left behind’ on the road towards UHC.

WHO is a health agency and so takes a public health approach to ACTA, an approach that seeks to support efforts towards UHC by identifying and addressing the underlying risk factors that increase the likelihood of health system corruption and create barriers to UHC. This is quite a different approach to traditional approaches to addressing corruption that have focused on prohibition, criminalization and punitive approaches. By taking a public health preventive approach to addressing the entry points where corruption could occur, much can be done to produce a paradigm shift in how health systems, as well as development partners, address ACTA. All societies and country contexts are vulnerable to corruption; acknowledging this, proactively building institutional capacity and ensuring measures to inhibit/prevent the development of corruption is an important part of wider reforms towards UHC. It enables the maximization of health benefits from public resources and builds public trust in the system. While punitive/remedial actions are often still required, an increased focus on prevention shifts the dominant focus from reactive measures towards creating innovations in prevention, including risk management, and opening new venues for addressing what is politically sensitive issue.

Health system corruption is a global public health problem and requires a global public health approach to solve it. To date, there has been an absence of a coordinated and coherent approach to ACTA amongst stakeholders working in global health, as well as between those working on ACTA at cross-sectoral levels and the health community both globally and domestically. This has limited the potential contribution of ACTA work to UHC. To respond to this challenge, work is underway to initiate a ‘Global network on ACTA in the health sector’. On 26–28 February 2019, a consultation was jointly convened by WHO, Global Fund and UNDP (with co-funding from UK Aid), with more than 130 global stakeholders focused on the goal of informing the workplan of this network. The consultation provided content for core network workstreams during the 2020–2023 period:

1. Rationalizing internal control and assurance models in health systems using fraud and corruption risk assessment methodologies;
2. Monitoring and evaluation of ACTA measures for health;
3. Capacity development on ACTA in the health sector for multiple stakeholders; and,
4. Integration of ACTA into health systems strengthening normative guidance.

The work of this network and the importance of ACTA to efforts towards UHC was highlighted at the UN High Level meeting on UHC in New York on 23 September 2019. This special issue provides a timely input to further discussions about the importance of ACTA started at the United Nations and makes a major contribution to WHO’s work on ACTA and to the four workstreams that are being developed for the global ACTA network. Specifically, this special issue contributes to all these efforts across five thematic areas:

## ACTA frameworks

The critical review by Vian [2] summarizes evidence on anti-corruption, transparency and accountability concepts, frameworks, and approaches. Vian identifies six typologies and frameworks which model relationships among factors influencing corruption, including transparency, accountability, and civic participation. Her article also identifies important approaches to anti-corruption, focused on strategies such as risk assessment, transparency interventions, audit, and systems-level reforms.

## ACTA and human rights

Sharifah Sekalala and co-authors examine corruption through a human rights lens [3]. They examine how human rights mechanisms can facilitate accountability for health services, thereby reducing the impact of corruption on the right to health. They discuss the empowerment of civil society actors, and how facilitating community representation in health policy and programmatic decision-making can help address corruption.

## ACTA and the health workforce

The review by Monica Kirya examines how corruption affects the recruitment and promotion of health workers and how this in turn affects access, quality and health outcomes [4]. Kirya analyzes global evidence related to six types of corrupt practices in recruitment and promotion, including patronage and clientelism, nepotism, cronyism, bribery, extortion and sextortion (a practice where sex, not money, is the currency of extortion or bribery). Kirya discusses how support is needed to help countries design and implement merit-based recruitment systems for human resources for health. This will not only ensure that properly qualified and skilled health workers attend to patients, but it is also as a means to curb other types of corruption in the health sector.

## Corruption and procurement

Kohler and Dimancesco argue for the importance of integrating ACTA measures into governance for pharmaceuticals procurement [5]. Their article provides a primer on key factors, types, and examples of corruption in pharmaceutical procurement. It examines in deeper detail the overarching role of good governance in pharmaceuticals, and specific anti-corruption tools such as integrity pacts, price transparency, open contracting, and e-procurement. Tim Mackey and Raphael Cuomo analyze evidence from the medical, engineering, and computer science literature to determine what we know about digital solutions to improve transparency and accountability in medicines procurement and supply [6]. They focus in particular on e-procurement systems, machine learning approaches, and other digital solutions allowing the detection of fraud and abuse.

## Arguing for a paradigm shift in anti-corruption efforts

Hunter et al. describe how the UNDP developed and tested a new approach to ACTA in the health sector in the Arab Countires (this is a UNDP classification for describing the region. WHO has a different classification and calls it the Eastern Mediterranean Region). This can provide an entry point for broader good governance reforms [7]. The authors note that national ACTA strategies have included legal frameworks, anti-corruption agencies, investigation and ensuing enforcement, and awareness-raising. While such strategies promote integrity, they argue that they provide insufficient guidance for control of corruption at the sectoral level, and are insensitive to context. Wierzynska and co-authors provide another perspective on how the international community engages in ACTA in the health sector [8]. Building on the UNDP work, the authors propose a paradigm shift from compliance to risk management centered on events that could be most harmful to health services and outcomes. Applying this Fraud Risk Assessment methodology, the new anti-corruption formula can help identify risks in a wide set of activities, from petty to grand, involving functions such as financing, supervision, service delivery, and data collection. Their article describes the steps to apply this method.

## A call to action for sustained ACTA efforts

Moving forward, this special issue supports a call for action for sustained efforts to combat health system corruption and to support the efforts of the global network on ACTA. Much more needs to be done to address health system corruption if we are to achieve UHC. More research is required about the magnitude, scope, characteristics and consequences of corruption. Much is still hidden and unexplored. We also need research to better diagnose the causes of health system corruption, the factors that increase or decrease the risk of corruption, and the factors that can be modified to reduce corruption risk through health systems strengthening interventions. Finally, we need to share ACTA success stories, especially how to implement effective and promising ACTA interventions in a wide range of health settings. This will allow all countries to seize the SDG pledge of leaving no one behind by integrating ACTA into the development and health agendas allowing for an increase in health system efficiencies and effectiveness and addressing inequalities that drive poor health and ultimately help achieve health for all.

## Paper context

This article describes how the World Health Organization regards anti-corruption, transparency and accountability measures as critical to health systems strengthening for universal health coverage and achievement of the UN sustainable development goals. It introduces this special issue on anti-corruption, transparency and accountability in the health sector. The articles contained herein are intended to support efforts by member states and other stakeholders to strengthen anti-corruption, transparency, and accountability in the health sector.
